# Development of a Smartphone App for Informal Carers of People With Cancer: Processes and Learnings

**DOI:** 10.2196/10990

**Published:** 2019-04-11

**Authors:** Natalie Heynsbergh, Leila Heckel, Mari Botti, Seung Chul O, Patricia M Livingston

**Affiliations:** 1 School of Nursing and Midwifery Faculty of Health Geelong Australia; 2 Epworth HealthCare Melbourne Australia; 3 School of Psychology Faculty of Health Deakin University Geelong Australia; 4 Faculty of Health Deakin University Geelong Australia

**Keywords:** cancer, carer, smartphone, mobile applications, technology

## Abstract

**Background:**

There are few support systems available to informal carers who provide care to cancer patients. Smartphone apps have the capacity to reach large audiences and can provide information and support at a time convenient to carers.

**Objective:**

The aim of this study was to design a smartphone app prototype for carers of adults with cancer.

**Methods:**

A multiple-method design was used to develop a smartphone app. Current and past carers of people with any type of cancer were recruited from a public hospital, a private hospital, and a carer organization, who participated in either a focus group or phone interview. Carers answered questions about items to include in an app to address supportive care needs identified. Using carers’ feedback, a smartphone app was designed and tested.

Beta testing was conducted using a convenience sample of participants who completed scenarios to inform the app’s design, functionality, and usability. Scenarios were timed and marked as complete or incomplete. Participants completed a questionnaire about the usability of the app. Beta testing occurred in 2 stages—a paper-based version of the app and an app-based test using the participants’ preferred device. Alpha testing was completed internally to ensure the functionality of the app. Data were collected between May 2016 and August 2017.

**Results:**

A total of 33 carers participated in phone interviews and 12 in focus groups; their average age was 55 (SD 14) years, and 60% (27/45) were female. The majority of carers (76%, 25/33) had a positive attitude toward using smartphone apps. Carers noted that smartphone technology might improve their ability to seek information and support in managing their own health as well as the care needs of the person with cancer. Carers requested a variety of information and resources to be included in the app. Paper-based testing included the following: participants (N=10) were aged above 30 years (30%, 3/10), 30 to 49 years (30%, 3/10), and 50 years or above (40%, 4/10), and 60% (6/10) were male. Participants found the app user-friendly and pleasing in appearance.

App-based testing included the following: participants (N=10) were aged above 30 years (20%, 2/10), 30 to 49 years (30%, 3/10), and 50 years or above (50%, 5/10), and 50% (5/10) were male. Participants reported the app to be user-friendly and easy to navigate. The majority (60%, 6/10) of participants were unable to create a shortcut icon to add the app to the home screen of their phone.

**Conclusions:**

Carers highlighted the needed information and support to assist them during the caring period; they also reported having a positive attitude toward smartphone apps. The Carer Guide App is currently undergoing a pilot study to further test usability among carers of people with 1 cancer type.

## Introduction

### Background

Cancer is a significant issue worldwide with over 14 million people diagnosed in 2012 [1] and is estimated to account for 9.6 million deaths in 2018 [2]. Globally, US $1.16 trillion are spent on cancer every year [2]. The financial burden on health care systems has resulted in quicker discharge times for patients and increased the need for care to continue in the community [3]. In Australia, there are approximately 2.7 million informal carers who are not paid for the care they provide [4]. Informal carers are often family members who may have limited awareness and understanding about the disease to sufficiently meet the care needs of individuals [5]. As a result, physical, mental, social, and financial burdens are common among carers resulting in negative health outcomes and poor well-being [6].

Carers often neglect their own needs while looking after someone with cancer [7,8]. Face-to-face support through local medical and counseling services can be costly, time consuming, and inaccessible to carers who are unable to leave care recipients alone or live in remote areas [9]. Technology may provide a solution in addressing the needs of many carers.

Technology-based tools allow large audiences to have access to information and support networks when addressing specific health needs [10]. Smartphone apps allow individuals to access information and support at a suitable time when needed and in the privacy and comfort of their own home [9,10]. Recent trends have shown increasing availability of 4G internet connection worldwide [11], and by 2020, 70% of the population is expected to own smartphones [12]. Although these figures suggest that smartphone and roaming internet access is common, individuals use technology in varying ways; therefore, it is important to assess carers’ attitudes toward digital technology as a supportive tool. Existing cancer information and support helplines are not widely recognized or used among people affected by cancer, and carers only account for approximately 20% of people who initiate contact [13,14]. Web-based interventions have been found to be appropriate for use among carers and are accessible to a larger number of people [15]; however, they are not always available through smart devices, and this can limit carers’ ability to access support in times of need [16]. Previous studies have shown positive results for the use of smartphone apps across different circumstances including self-management of cancer [17,18], for carers of pediatric illness [19,20], and for children with cancer [21]. However, there have been no studies assessing the use of smartphone apps among adult carers providing care to another adult with cancer [15].

### Theoretical Frameworks

This research was guided by 2 theoretical frameworks. The theory of planned behavior (TPB) and the unified theory of acceptance and use of technology (UTAUT). TPB applies 3 concepts—behavioral beliefs, normative beliefs, and control beliefs for understanding social and personal reasons for using technology [22].

The concept “facilitating conditions” within UTAUT measures external factors contributing to technology use such as the ownership of a smartphone device and internet connectivity [23].

### User-Centered Design

User centered design (UCD) is a philosophy to guide the design of interventions to meet needs, preferences, and characteristics of users, using a lifecycle process of context, requirements, design, and evaluation [24,25]. UCD has been used to develop technology-based interventions among a variety of populations [26-28].

### Aim

The aim of this study was to design a smartphone app prototype for carers of adults with cancer.

## Methods

### Study Design

This study comprised a multiple-methods design to inform development of the app and included the following 3 sequential phases: (1) focus groups and phone interviews with present and past adult carers to assess their information and supportive care needs as well as their attitudes toward smartphone technology, including existing barriers affecting technology uptake; (2) smartphone app design, content development, and app programming; and (3) alpha and beta testing and user testing of the app. Findings from phase 1 informed the design of the app and its content. Data were collected between May 2016 and August 2017. Ethics approval was obtained from Deakin University Human Research Ethics Committee, from 2016 to 2018.

### Context of Use

The context of carers of people with cancer and their needs from a smartphone app were identified in phase 1 via focus groups and interviews. TPB and UTAUT guided the development of the app in terms of its structure and function (eg, font size and navigation) and accessibility to carers’ with varying skills and confidence in using smartphone apps. The design phase was completed by combining these results. Evaluation occurred during alpha and beta testing through paper-based and app-based user testing. Between paper-based and app-based testing, the needs of participants were identified, and the design solutions to match these needs were performed and evaluated in line with the UCD methodology.

### Specify Requirements: Integration of Theoretical Frameworks

TPB and UTAUT were incorporated into the development of the smartphone app across the different stages. See Table 1 for an outline of how the frameworks were applied to support development of the app.

**Table 1:** Theoretical frameworks and how they were used to support Carer Guide App development.

Framework, concept		Description	Relevance to app development
**Theory of planned behavior (TPB)**			
	Behavioral beliefs	Attitudes toward using smartphone apps	Participants were asked about their attitudes toward using smartphone apps during focus groups and interviews. Participants in phone interviews responded with positive, neutral, or negative attitude toward smartphone apps. The overall group consensus was reached in focus groups.
	Normative beliefs	People who may facilitate or create a barrier toward smartphone app use	Participants in phone interviews provided information about who would facilitate their use of a smartphone app, for example, health care professionals, family, or friends. The overall group consensus was reached in focus groups.
	Control beliefs	Confidence in using smartphone apps	Participants were asked about their confidence in using smartphone apps during focus groups and interviews and user testing during development. Participants in phone interviews responded with very confident, moderately confident, or novice. The overall group consensus was reached in focus groups.
**Unified theory of acceptance and use of technology (UTAUT)**			
	Facilitating conditions	External factors that may be a barrier to using smartphone apps	Measured during focus groups and phone interviews, participants gave information about factors that affected their likelihood of using smartphone apps, for example, smartphone ownership.

### Creating Design Solution

#### Phase 1: Focus Groups and Phone Interviews

To develop a smartphone app that was responsive to carers and specific to their needs, focus groups and phone interviews were conducted with current and past adult carers looking after another adult with cancer of all types and stages. Carers were invited to participate if they were older than 18 years and able to speak English sufficient to participate in the group discussion. Questions explored the attitudes (behavioral beliefs), facilitating influences (social norms), confidence (control beliefs), facilitating conditions affecting smartphone app use, and the content desired in a smartphone app to address carers’ needs. Demographic data including age, gender, relationship to patient, highest level of education, and living situation were collected. The majority of participants (80%, 36/45) provided information about the type of cancer their family member or friend was diagnosed with. Recruitment continued until saturation of data occurred; overall, 45 carers were recruited (12 into focus groups and 33 into phone interviews).

#### Phase 2: Design, Content Development, and Programming of the App

**Figure 1:**
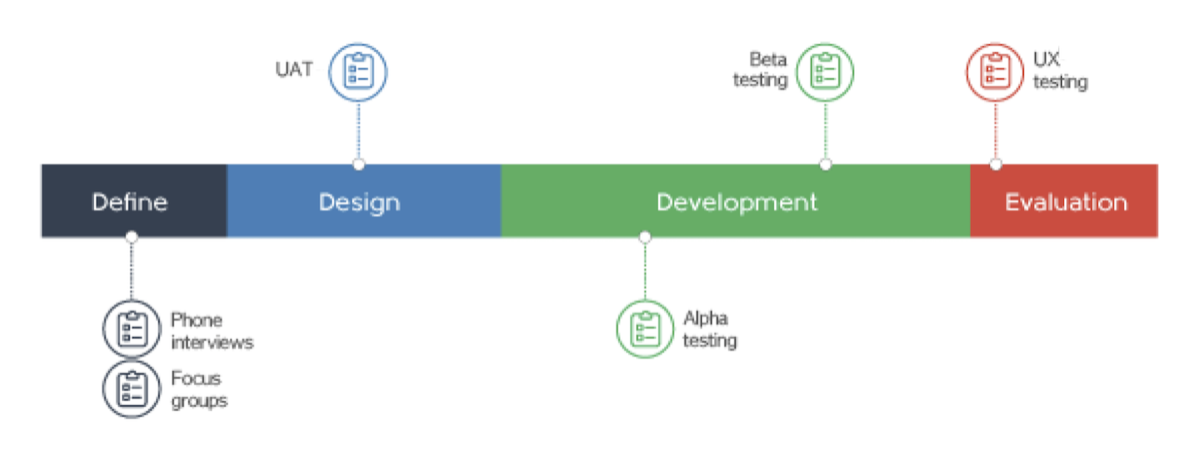
Development stages of the Carer Guide App. UAT: user acceptance test; UX: user experience testing.

The smartphone app, referred to as the Carer Guide App, was designed to support carers of people with cancer based on Shneiderman’s “Eight Golden Rules of Interface Design.” The content and high-level user experience were informed by the findings of phase 1 of the project. The Carer Guide App was designed and developed by e-Resource developers at Deakin University and built using a hybrid Web-based structure, incorporating technologies including—Adobe Illustrator CC, Adobe Photoshop CC, HTML 5, CSS 3, JavaScript, JQuery, Ajax, PHP, and MySQL (Oracle). Email notifications to users were triggered by a time-based scheduler (known as a cron job) in a Unix-like computer operating system. A hybrid Web-based structure was chosen over that of a native app as it significantly reduced time required in the development stage of the project, including programming, updating functionality, and content revision. Further, the structure that was chosen did not require distribution through either the App Store or Google Play. This saved time in deployment as the often-lengthy review processes of those distribution channels were bypassed. The chosen structure allowed development of the app, which was accessible on a wider range of devices. The app was accessed at a URL address through any current generation mainstream internet browser. The app contained both static and dynamic content accessible through a primarily iconized navigation system. The text in the app contained links to both external websites and built-in interactive functionality to maximize user experience. Security of sensitive information provided by users was a priority, enhanced by features such as personalized secure log-ins and encrypted data. The Carer Guide App took 3 months to develop including user testing and alpha and beta testing. Figure 1 outlines the stages of the app development process.

#### Phase 3: Evaluate Designs—Testing of the App

##### Paper-Based User Acceptance Test

**Figure 2:**
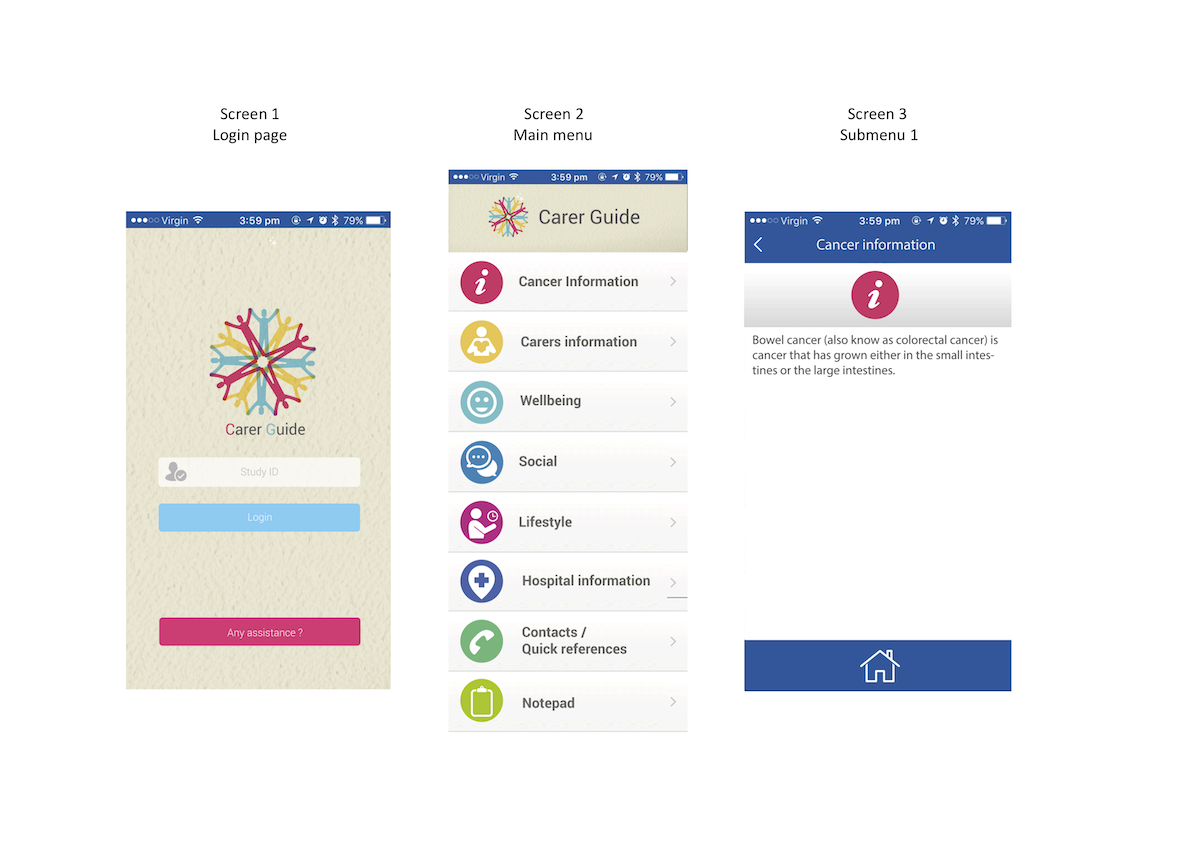
Screenshots of the Carer Guide App used in the paper-based user test.

A convenience sample of 10 adults was recruited to test a paper-based version of the Carer Guide App before the development of the prototype. Paper-based testing was conducted to assess the visual elements of the app and initial content layout and navigation. This was achieved using printed screenshots of the app. Figure 2 presents an example of screenshots used—screen 1 was the log-in page, screen 2 was the main menu, and screen 3 was the relevant information page.

During UAT, participants were asked to complete scenarios in which they had to navigate the app to locate information, for example, “You require information about financial aid, where would you go to learn about benefits you are entitled to?” Participants also completed a questionnaire including information about—their gender, age, confidence in using apps (control beliefs), usability of the app, and comments for improvement.

##### Alpha and Beta Testing

The Carer Guide App underwent several rounds of internal testing known as alpha and beta testing [29]. Alpha testing was used to assess the input and output of the functions of the app and was performed by the developer while building the structure of the app [29]. Beta testing assessed the complete function and applicability of the app using a smartphone interface among test participants [29]. Google Analytics was linked to the Carer Guide App to collect usage information on—the number and length of sessions, device used, and frequency of pages visited from each participant.

##### Beta Testing: App-Based User Testing

A second convenience sample of 10 adults was recruited to test the first prototype version of the Carer Guide App. Test participants were asked to complete scenarios in which they had to download the app, create a shortcut icon, log into the app, navigate to locate information, access hyperlinks and phone numbers, and navigate through website browsers. Participants also completed a questionnaire including information about their gender, age, confidence in using apps (control beliefs), functionality of the app, and comments for improvement.

### Data Analysis

#### Focus Groups and Phone Interviews

Data from focus groups and phone interviews were transcribed and coded. A qualitative descriptive approach was used to analyze data [30], and the full analysis procedure has been described in more detail elsewhere [31]. Items suggested by carers to be included into a smartphone app were organized into common categories, for example, information about cancer treatment and information about side effects were grouped together under “cancer information.” The frequency of suggestions for app content from carers participating in phone interviews was tallied. Focus group data were analyzed by general group consensus where carers discussed and agreed on concepts; items suggested to be included in the app were organized into the same categories as phone interviews. Data were coded and analyzed using the NVivo (QSR International) software.

To assess theoretical framework measures, the frequency of responses from carers in phone interviews was tallied. Data from focus groups were analyzed by overall group consensus. Data were analyzed using IBM SPSS software.

#### User Acceptance Test and User Experience Testing

In UAT testing, usability of the app was measured on a Likert scale where 1 meant strongly disagree and 5 meant strongly agree. In UX testing, a similar scale was used to assess functionality of the Carer Guide App. Agree and strongly agree responses were then tallied.

Scenarios were timed and organized into 2 groups: those taking less than 20 seconds to complete, and those taking longer than 20 seconds to complete. This was determined to be an appropriate cut-off time as Web users often only stay on pages for 10 to 20 seconds when seeking information [32]. To ensure the organization of information was relevant, 20 seconds was deemed an appropriate amount of time to navigate and locate information.

In each round of user testing, participants described their level of confidence in using smartphone apps by selecting 1 of 3 options—very confident, moderately confident, or not confident. Responses were then tallied. Data were analyzed using the SPSS software.

## Results

### Focus Groups and Phone Interviews

The majority of carers were female (60%, 27/45), a spouse (64% 29/45), living with the person receiving cancer treatment (87%, 39/45), held a university degree (47%, 21/45), and caring for someone with breast cancer (30%, 11/45). Carers age ranged from 21-80 years (SD 14) with mean age of 55 years. See Table 2 for full demographic information.

**Table 2:** Demographic characteristics of carers.

Carer characteristics		Frequency n (%)
Female		27 (60)
**Carers relationship to patient**		
	Spouse	29 (64)	
	Parent	13 (29)
	Other (relative or friend)	3 (7)
Lives with patient		39 (87)
**Highest education level**		
	Primary school	1 (2)
	High school	9 (20)
	Certificate or Diploma	7 (16)
	University degree	21 (47)
	Other	6 (13)
**Patients’ cancer diagnosis as reported by carers**		
	Breast	11 (30)
	Lymphoma or non-Hodgkin’s lymphoma	7 (19)
	Pancreas	3 (8)
	Leukemia	3 (8)
	Liver	2 (6)
	Lung	2 (6)
	Colorectal	2 (6)
	Other (eg, brain, prostate, stomach, multiple myeloma, bone, and neck)	69 (17)

Carers provided varied ideas for content that could be included in the app. Overall, carers reported a need for more cancer-related information, links to support services and social networks, case studies, interventions to manage symptoms at home, information on how to identify serious side effects, and when to escalate care, on hospital-specific navigation, and resources to manage their own needs. Resources mentioned included—calendar with symptom tracking, reminders for appointments and medications, notepad, contacts, a search function, and the ability to synchronize the app with other phone functions. Carers specified that the app should have information specific to their needs and the use of push notifications was regarded as beneficial because the app would be perceived as less impersonal.

Some carers felt that more than 1 person could facilitate their use of a smartphone app. Overall, 15% (5/33) carers would not be influenced by others to use an app and were more likely to prefer using the computer or talking face-to-face with a health care professional. Refer to Table 3 for a full summary of results related to theoretical frameworks and their implementation into practice.

### Design, Content, and Technical Development

**Figure 3:**
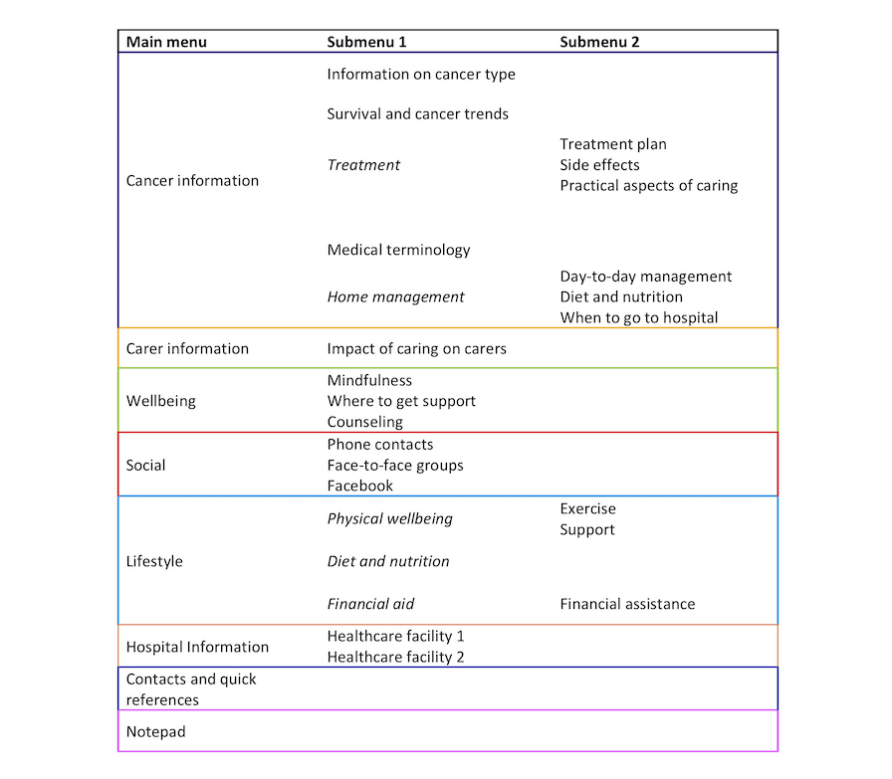
Initial structure of the Carer Guide App.

Results from phase 1 indicated that carers required the app to be specific to their information and support needs. Initial development decisions included—app name, color scheme, logo and icon pictures, and the layout structure. To ease navigation, similar content materials were grouped together under 1 main category; this is shown in Figure 3.

### Testing of the Carer Guide App

#### User Acceptance Test

The sample of 10 included past carers, noncarers, and a medical professional on an oncology ward. Participants’ age ranged above 30 years (30%, 3/10), 30 to 49 years (30%, 3/10), and 50 years or above (40%, 3/10); 60% (6/10) were male. Confidence in using smartphone apps is outlined in Table 2.

**Table 3:** Results related to theoretical framework concepts and their implementation into practice.

Framework	Focus groups and phone interviews	User acceptance test	User experience testing	Implementation into practice
Behavioral beliefs	Focus group consensus was positive toward smartphone app use. Overall, 76% (25/33) participants from phone interviews had a positive attitude toward using smartphone apps.	—^a^	—	A smartphone app may be an appropriate way to deliver information and support to carers. Carers’ attitudes toward the Carer Guide App in particular need further assessment to provide more information about the suitability of a supporting smartphone app.
Normative beliefs	Participants in phone interviews felt smartphone apps could be facilitated by the following: health care professionals (79%, 26/33); social networks (21%, 7/33); anyone (9%, 3/33); cancer organizations (6%, 2/33); others in the same situation (6%, 2/33). app store listings (3%, 1/3).	—	—	Dissemination of a smartphone app may best be supported by health care professionals. This needs more investigation.
Control beliefs	Overall, participants in focus groups were confident in using smartphone apps. In phone interviews, 82% (27/33) participants were confident with using smartphone apps. Participants with lower confidence were infrequent or nonusers.	Overall, 60% (6/10) participants were very confident in using smartphone apps, 30% (3/10) were moderately confident, and 10% (1/10) were novice.	Overall, 50% (5/10) participants were very confident in using smartphone apps, and 50% (5/10) were moderately confident. Participants stated instructions or a guide to using the Carer Guide App would improve their confidence.	Video instructions were developed to aid carers using the Carer Guide App.
Facilitating conditions	Overall, 9% (3/33) participants in phone interviews noted barriers to smartphone app use included not owning a smartphone, not using smartphone apps, and not having adequate internet connection at home. The overall consensus from focus groups identified lack of smartphone ownership as a barrier; this was among a minority.	—	—	A smartphone app may be a relevant way to deliver information and provide support to carers as the majority of the sample experienced no impact of facilitating conditions. Facilitating conditions are likely to reduce as more people continue to use smartphones.

^a^These concepts were not measured during this phase of development.

Overall, the appearance and layout of the Carer Guide App were considered favorable; however, there was some confusion between 2 icons, *Lifestyle* and *Wellbeing*. Of the 16 tasks, 13 were completed successfully by 100% of the participants. The 3 tasks not completed by all participants included—finding financial aid (60%, 6/10 completed), seeking counseling sessions (90%, 9/10 completed), and seeking peer support (90%, 9/10 completed). These 3 topics related to the app icons— *Lifestyle, Wellbeing*, and *Social*. The time taken by participants to complete 5 scenarios was as follows—finding financial aid (66.5 seconds), counseling sessions (36.8 seconds), seeking peer support (26.1 seconds), finding carer resources (29.7 seconds), and saving and exiting the notepad (26 seconds).

Overall, the Carer Guide App’s features and functionalities were satisfied by the testing group. Out of a score of 5, participants found the app was easy to navigate and visually appealing (5 out of 5). The icon pictures were also relevant to information on the individual pages. Participants were asked for suggestions to make the app more user-friendly; participants suggested changing the iconized navigation titles, rearranging the layout of contents, and having the capabilities to synchronize app content with other phone functions.

The following UAT changes were incorporated to improve the appearance and usability of the Carer Guide App:

1. The icons *Lifestyle* and *Wellbeing* were merged. Icon name— *Wellbeing*. Icon picture—smiley face. Icon contents—physical well-being, diet and nutrition, counseling, and mindfulness activities.
2. A separate financial aid icon was created. Icon name— *Financial and legal*. Icon picture—dollar symbol.
3. The icon *Social* was renamed to specify that it relates more to connecting with others rather than social issues, for example, social work. Icon name— *My social network*.
4. The icon *Contact/quick references* was renamed to reduce ambiguity. Icon name— *Contacts*.

**Figure 4:**
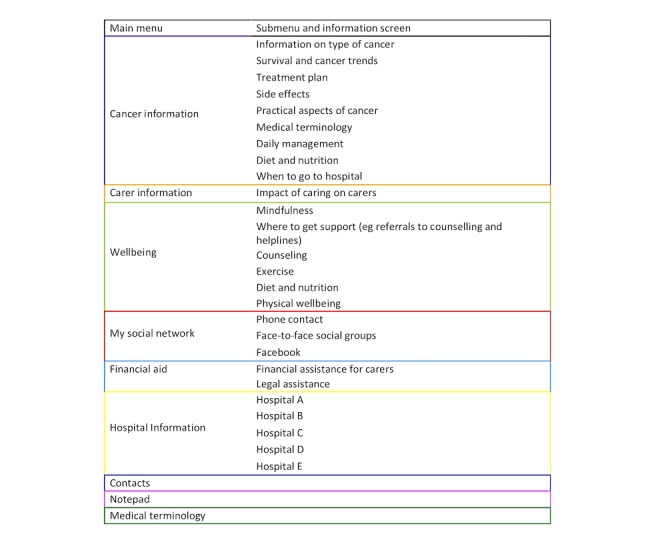
Modified structure of the Carer Guide App.

Following UAT, the layout of the main menu and submenus were modified and are displayed in Figure 4. Submenus 1 and 2 were condensed after consultation with the app developer to reduce the number of screens participants would have to search through to find the information required. During this process, *Medical Terminology* was included in both the main menu and the *Cancer Information* submenu. This was done as the content was relevant for inclusion in *Cancer Information*, but because of the volume of information in this section, it may have required additional time to access. To enhance usability, *Medical Terminology* was included in the main menu of the Carer Guide App for quick reference use.

#### User Experience Testing

An equal number of females (n=5) and males (n=5) tested the app. Participants were younger than 30 (30%, 3/10), 30 to 49 years (20%, 2/10), and 50 years or above (50%, 5/10). The sample included past carers and noncarers. Android operating systems were used by 30% (3/10) of people, and iPhone operating system (iOS) was used by 70% (7/10). Confidence in using apps is outlined in Table 2.

Of the 23 tasks, 18 were completed by 100% of participants. Completion rates for the following 5 tasks were lower: creating a shortcut icon (40%, 4/10 completed), finding peer support (90%, 9/10 completed), adding a new contact (90%, 9/10 completed), returning to the Carer Guide App window after visiting an external website (90%, 9/10 completed), and clicking on an external number to make a call (90%. 9/10 completed). Overall, 4 of these tasks related to system factors and 1 related to misunderstanding of content.

On average, participants completed the majority of tasks (18 out of 23 tasks) in less than 20 seconds. Overall, 5 tasks took participants on average greater than 20 seconds to complete; this included downloading the app (31.8 seconds), creating a shortcut icon (23 seconds), finding symptom management (46.5 seconds), finding benefits and payments (20.7 seconds), and seeking peer support groups (29.3 seconds).

Participants rated the app as easy to use, and the phone numbers were clear and easy to recognize and access (4.7 out of 5 for each aspect). Ease of accessing the app after visiting an external website was scored 4.1 out of 5. The highest usability factors of the app were awareness of external website links and ease of accessing external links; these scored 4.9 out of 5, and corresponding tasks were completed by 100% (10/10) of participants. Ease of creating a shortcut icon was the lowest scoring aspect (3.6 out of 5), as most participants (60%, 6/10) were unable to complete this task. Of the 6 participants who could not create the app icon shortcut, 2 still rated it 3 out of 5 as they stated it was easy to do once shown.

The Carer Guide App was tested on both Android and iOS devices to assess any variation in the performance of the app. During testing, it was noted that there were differences between the operating systems. On Android devices, problems encountered included not being able to find the shortcut icon once created, internet and phone links not connecting to external sites or numbers, and the “Add” button in the contact menu did not appear. On iOS devices, icon pictures were enlarged, and pictures appeared in incorrect menus. These errors were not present among all iOS versions. Android errors occurred for 2 participants, and iOS errors occurred for 1 participant.

Comments for improvement included instructions to create a shortcut icon and improvements in system factors, for example, working links and phone numbers. Individual participants requested changes to iconized navigation titles, layout such as having items in menu format, and the ability to synchronize app features to phone features.

On the basis of these test results, the following steps were taken to improve the Carer Guide App:

1. Confirm all links, pictures, and buttons are correct and working in all operating systems.
2. Inclusion of instructional downloading and navigation videos for both iOS and Android operating systems, comprising information on how to create the shortcut icon, how to navigate between different browsers, how to close browsers, highlight weblinks and phone numbers, and how to use them.

## Discussion

### Principal Findings

Caring for someone with cancer can be stressful, and information and support are not easily available [33,34]. The Carer Guide App was developed to support carers while caring for someone with cancer. Carers may be reluctant to communicate their own needs and struggle to find information that is specific to their own situation [35]. The Carer Guide App provides a means for carers to access information and support anywhere within their internet connection capabilities and allows carers privacy in addressing their needs [9].

The development followed a co-design process, which sought involvement from stakeholders throughout the design and creation phase of development [36]. Involving carers in the creation of the Carer Guide App enabled the content to be designed specifically for carers’ needs. The sample was a heterogeneous group, with participants caring for people with different types of cancer, of different ages, and various stages of caring including new, ongoing, recurrent, or past carers. This allowed the Carer Guide App to be designed to address the needs of carers from a variety of clinical, demographic, and social perspectives. Involvement of stakeholders in the development of technology-based interventions is an important part of UCD to ensure systems match users’ needs [25]. Using interviews to learn about stakeholders needs have been used among a variety of different groups including people with mental illness [37], among parents and teenagers with asthma [38], and for improving physical activity among people with chronic illnesses [39]. These studies found similar results, suggesting that intervention content should be highly relevant to stakeholders' needs, and in an easy-to-use format [37,38]. During user testing, inclusion of noncarers was important as not all people have previous experience with cancer before becoming a carer. This allowed the Carer Guide App to be tested among people with no previous knowledge of how to address cancer-related needs. Both the UAT and the UX testing showed that participants found the appearance of the Carer Guide App favorable. Issues with navigation during UAT were amended, and participants in the UX test were more easily able to navigate the Carer Guide App. Results from the UX test highlighted the need for specific instructions to accompany the Carer Guide App. UX has been used in the literature to capture design and navigation flaws before larger trials or integration into practice [18]. Tying in with the theoretical frameworks, the Carer Guide App was used successfully among people with varying levels of confidence. Feedback during phases 1 and 3 demonstrated participants’ positive attitudes toward the development of the Carer Guide App. Factors potentially affecting Carer Guide App usage included recommendations from health care professionals to use the app. The influence of health care professionals on carers’ information-seeking behavior is consistent with findings from previous research [40-42] and highlights the need to involve staff working in oncology settings in the implementation process for new interventions or services. Barriers to using apps included not having access to a smartphone or the internet; however, this only affected 9% (3/33) of this sample. Furthermore, smartphone ownership is expected to increase over time, suggesting smartphone apps are a relevant way to deliver resources to carers [12].

The concepts from the theoretical frameworks were easy to measure and relevant to the development process and could be implemented in any stage of the project. Findings from the theoretical framework provided the study with baseline results about the appropriateness of a smartphone app for carers of people with cancer and highlighted potential dissemination methods, for example, health care professionals to guide future research.

### Challenges Encountered

Although participants were engaged during the development of the Carer Guide App, it was not possible to meet the requests of all carers. For example, requests to include interactive features may affect the overall usability for carers who may be less confident in using apps. As a result, interactive features such as discussion boards and symptoms trackers were not created in this version of the app, and synchronizing features were not included in the app.

A second challenge was creating the app within the time frame of the research project. The Carer Guide App was developed as a Web-based app. Although Web-based apps are quicker to develop and launch and easier to modify, it required a different approach to downloading the app. Using the Web-based app, participants were required to create a shortcut icon and navigate through browser windows when external links were accessed from the Carer Guide App. These factors required testing during the UX test to assess whether participants could understand and navigate these factors and identified the need to develop video instructions for carers to assist them in completing these tasks. However, development of a Web-based app allowed secondary analyses to occur to assess which devices carers used on the Carer Guide App, for example, a phone, tablet, or computer; this may allow for an in-depth analysis about the applicability and acceptance of smartphone apps among carers.

### Strengths of the Study

To the researchers’ knowledge, this app is the first of its kind as carers guided its development, including the content, visual presentation, and layout. This research used a co-design process by involving carers (as stakeholders) during each phase of development and seeking user feedback to improve system functionality. This approach may be useful for future research to guide the development of novel interventions. Another strength of this research was the inclusion of current carers of a variety of cancer types and stages as well as past carers during focus groups and phone interviews. This enabled the content of the app to be created to meet the needs of carers across the illness trajectory.

### Limitations

This study has several limitations including the collection of information from carers living in metropolitan areas only, who spoke English. This may have resulted in the development of an app that is not appropriate for carers living in rural and remote areas or who speak a primary language other than English, as they may experience different needs. The Carer Guide App was not designed to synchronize to other phone functions because of the need to incorporate additional security measures. Not synchronizing the Carer Guide App to phone functions decreased the need for security passwords to access the app; this reduced any burden of having to remember passwords in times of stress by recipients.

Interactive features such as symptom tracking and calendars were not incorporated into this version of the Carer Guide App as they required an extended amount of time to create and test. When developing interventions with interactive features or the ability to synchronize to other phone functions, future researchers should consider the development time frame of their intervention, including the time needed to launch apps through Google Play and the App store.

### Recommendations for Future Research

Future research is needed to assess the applicability of apps for carers living in rural and remote areas and those whose primary language is not English. These groups of people may experience different needs and therefore require other information and services within an app.

### Next Steps

On the basis of the development of the Carer Guide App outlined in this study, a pilot study is assessing the feasibility, usability, and acceptability among carers looking after people with 1 type of cancer. Findings from the pilot study will complete the UCD process by providing information about the suitability of the Carer Guide App among this population.

### Conclusions

In conclusion, carers require information and support during the caring period. A smartphone app may provide 1 solution to address these needs. A pilot study is currently underway to test the feasibility, usability, and acceptability of the Carer Guide App.

## Abbreviations

iOS: iPhone operating system
TPB: theory of planned behavior
UAT: user acceptance test
UCD: user-centered design
UTAUT: unified theory of acceptance and use of technology
UX: user experience testing
